# Towards a Deeper Understanding: Utilizing Machine Learning to Investigate the Association between Obesity and Cognitive Decline—A Systematic Review

**DOI:** 10.3390/jcm13082307

**Published:** 2024-04-16

**Authors:** Isabella Veneziani, Alessandro Grimaldi, Angela Marra, Elisabetta Morini, Laura Culicetto, Silvia Marino, Angelo Quartarone, Giuseppa Maresca

**Affiliations:** 1 Department of Nervous System and Behavioural Sciences, Psychology Section, University of Pavia, Piazza Botta, 11, 27100 Pavia, Italyalessandro.grimaldi01@universitadipavia.it (A.G.); 2 IRCCS Centro Neurolesi “Bonino-Pulejo”, S.S. 113 Via Palermo C. da Casazza, 98124 Messina, Italy; angela.marra@irccsme.it (A.M.); elisabetta.morini@irccsme.it (E.M.); silvia.marino@irccsme.it (S.M.); angelo.quartarone@irccsme.it (A.Q.); giusy.maresca@irccsme.it (G.M.)

**Keywords:** cognitive decline, obesity, BMI, machine learning, artificial intelligence

## Abstract

**Background/Objectives**: Several studies have shown a relation between obesity and cognitive decline, highlighting a significant global health challenge. In recent years, artificial intelligence (AI) and machine learning (ML) have been integrated into clinical practice for analyzing datasets to identify new risk factors, build predictive models, and develop personalized interventions, thereby providing useful information to healthcare professionals. This systematic review aims to evaluate the potential of AI and ML techniques in addressing the relationship between obesity, its associated health consequences, and cognitive decline. **Methods**: Systematic searches were performed in PubMed, Cochrane, Web of Science, Scopus, Embase, and PsycInfo databases, which yielded eight studies. After reading the full text of the selected studies and applying predefined inclusion criteria, eight studies were included based on pertinence and relevance to the topic. **Results**: The findings underscore the utility of AI and ML in assessing risk and predicting cognitive decline in obese patients. Furthermore, these new technology models identified key risk factors and predictive biomarkers, paving the way for tailored prevention strategies and treatment plans. **Conclusions**: The early detection, prevention, and personalized interventions facilitated by these technologies can significantly reduce costs and time. Future research should assess ethical considerations, data privacy, and equitable access for all.

## 1. Introduction

Obesity is a condition characterized by the excessive accumulation of body fat and a body mass index (BMI) of 30 or higher, in relation to lean mass [1]. In 2022, over 2.5 billion adults aged 18 and above worldwide were overweight, with 890 million of them having obesity, making it a global public health crisis [2]. In Italy, the picture is equally concerning, with 35.5% of the adult population overweight and 10.8% obese [1]. The economic consequences of the obesity epidemic are also significant. Without intervention, it is estimated that the global costs of overweight and obesity could reach USD 3 trillion annually by 2030 and over USD 18 trillion by 2060 [3]. This alarming trend carries significant health implications, as obesity is a major risk factor for numerous chronic diseases, namely heart disease due to high blood pressure and high cholesterol levels, type 2 diabetes, and breathing problems such as asthma and sleep apnea [4]. In 2019, an estimated 5 million deaths from noncommunicable diseases (NCDs) were caused by higher-than-optimal BMI, including neurological disorders [5]. The societal impact of obesity extends beyond healthcare costs and individual health outcomes. Obese individuals may face discrimination in employment, social settings, and even the healthcare system. This can lead to feelings of isolation, depression, and decreased quality of life [6]. Additionally, the rise in obesity places a strain on public health infrastructure and resources, diverting funds from other preventative and treatment programs [7]. Addressing the obesity epidemic requires a multi-pronged approach, including public health initiatives to promote healthy eating and physical activity, improved access to affordable and nutritious food options, and addressing the social and environmental factors that contribute to weight gain [8]. Furthermore, recent research has shown a concerning link between obesity and cognitive decline, adding another layer of complexity to this multifaceted public health challenge [9].

Cognitive decline occurs when an individual’s cognitive abilities, such as memory, attention, and reasoning, deteriorate faster than what is considered normal for their age and condition. It can arise from various factors, such as aging, neurodegenerative diseases, and brain injuries [10]. Dementia, which is also referred to as a “major neurocognitive disorder”, affects cognitive, behavioral, mood, and personality functions [11]. As a result, there is a significant change in the patient’s functional state, which impacts their independence and relationships with others [12]. The global burden of cognitive decline mirrors that of obesity. Over 55 million individuals worldwide are estimated to be living with dementia, with this number projected to double every 20 years [13]. In Italy alone, there are over 1 million patients with dementia (of which about 600,000 have Alzheimer’s dementia) and about 3 million people involved in their care. This has consequences on the economic and organizational plan [14]. The estimated worldwide cost of dementia was USD 818 billion in 2015, which represented 1.09% of the global gross domestic product (GDP) at that time. The annual global cost of dementia is now above USD 1.3 trillion and is expected to rise to USD 2.8 trillion by 2030 [13].

The link between obesity and cognitive decline is particularly concerning. Obesity doubles the risk of Alzheimer’s disease (AD) and is linked to a higher likelihood of dementia [15,16]. A recent study has shown that individuals who are overweight or obese have a higher risk of developing dementia compared to those within a normal weight range [17]. For those with a BMI between 25 and 29.9, the risk of developing dementia is increased by 27%, while those with a BMI of 30 or higher have a 31% increased risk [18]. Postmortem studies show that specific proteins associated with AD (β-amyloid and tau proteins) were higher in elderly people with severe obesity [19]. Higher BMI predicts temporal lobe atrophy, and there is evidence that obesity also increases the risk of mild cognitive impairment (MCI), a precursor to dementia [19,20,21,22]. The reasons for this connection are many and complex. Research suggests that obesity can accelerate brain aging by up to 16 years, shrink brain volume, and weaken the brain’s resilience to damage, leading to worse symptoms and faster disease progression [23,24,25,26]. Metabolic syndrome (MetS) involves a cluster of medical conditions such as insulin resistance, type 2 diabetes, dyslipidemia, and AD, sometimes dubbed as type 3 diabetes [27]. Exposure to environmental estrogens and anti-androgens can interfere with normal pancreatic function, insulin signaling pathways, brain insulin resistance, and dyslipidemia, increasing the risk of developing diabetes and related dementia [28]. This connection hinges on a key molecule: insulin. Insulin regulates blood sugar levels and plays a vital role in brain function and memory. If the body becomes resistant to insulin, a key feature of type 2 diabetes, it can disrupt the brain as well, possibly leading to cognitive impairment and increasing the likelihood of Alzheimer’s and other neurodegenerative diseases [29]. Dyslipidemia and elevated levels of triglyceride-rich lipoproteins (TRLs) in type 2 diabetes can combine and bind to amyloid-β peptide (TRL-Aβ), which can damage cerebral capillary integrity, leading to neurovascular inflammation, neuronal damage, and premature cognitive decline [30]. People can reduce their risk of cognitive decline and dementia by being physically active; not smoking; avoiding the harmful use of alcohol; controlling their weight; eating a healthy diet; and maintaining healthy blood pressure, cholesterol, and blood sugar levels [12]. By adopting these healthy lifestyle habits, individuals can not only improve their physical health but also potentially protect their cognitive function and reduce their risk of dementia later in life.

Given the immense societal and personal costs associated with both obesity and cognitive decline, the need for innovative strategies to prevent and treat these conditions is paramount. Artificial intelligence (AI) and machine learning (ML) are promising tools that can revolutionize research in this area [31]. AI is a field of computer science that creates machines and software capable of performing tasks that typically require human intelligence [32]. ML is a branch of AI that uses algorithms and statistical models to enhance performance through data analysis [33,34]. In clinical practice, AI and ML analyze datasets to identify new risk factors, construct predictive models, and develop customized interventions, providing valuable insights to healthcare professionals [32,33,34,35]. This deeper understanding of the underlying mechanisms can pave the way for more effective prevention strategies [36]. Beyond risk prediction, AI and ML offer exciting possibilities for personalized interventions. These models can utilize AI and ML algorithms to analyze individual data and predict the risk of developing obesity or cognitive decline [37]. Early risk identification allows for timely interventions, potentially mitigating the severity and progression of these conditions [38]. AI and ML can also be used to create personalized interventions tailored to individual needs and risk factors [32]. For instance, an AI-powered app can analyze a user’s dietary habits, physical activity levels, and genetic data to identify their risk for obesity and cognitive decline. The app could then recommend personalized dietary plans that promote healthy eating and weight management. It could also suggest exercise programs tailored to the user’s fitness level and preferences while incorporating cognitive training activities to stimulate brain function. The potential benefits of such personalized interventions are multifaceted. A recent systematic review and meta-analysis demonstrated that chatbots can motivate individuals to increase their daily step count, consume more fruits and vegetables, and improve sleep duration and quality [39]. Furthermore, chatbots for supporting hypertension medication self-management were found useful in managing patient’s medications, including reminders, refills, and even communication with healthcare providers [40]. These results overall suggest chatbots as a promising tool for improving self-management and medication adherence in patients with hypertension. By addressing the root causes of obesity and cognitive decline in a targeted manner, these interventions can lead to improved health outcomes and a reduction in healthcare costs. As previously observed, these personalized approaches can empower individuals to take control of their health and well-being, fostering motivation and adherence to lifestyle changes. By continuing to invest in research and development in this field, we can unlock the full potential of these technologies to create a future where these debilitating conditions can be effectively prevented, managed, and even potentially reversed.

Therefore, this systematic review aims to evaluate the application of AI and ML techniques in research related to obesity, its associated health consequences, and cognitive decline. This review can provide valuable insights into the current state of the art and pave the way for future advancements in this crucial area of public health.

## 2. Materials and Methods

**Figure 1:**
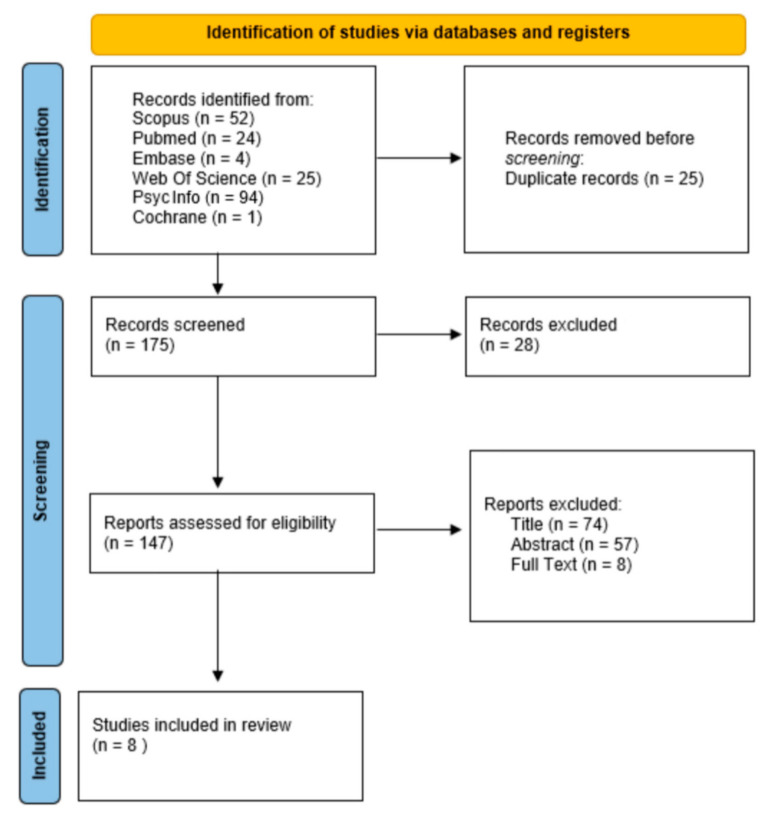
Prisma flow diagram for research strategy.

This systematic review was conducted and reported in accordance with the Preferred Reporting Items for Systematic Review and Meta-Analyses (PRISMA) (see Figure 1). A protocol for this systematic review was established and preregistered on the Open Science Framework (OSF) on 13 March 2024 (N2CGK).

### 2.1. Search Strategy

The search for articles was conducted in January 2024. Articles were selected from research databases—PubMed, Cochrane, Web of Science, Scopus, Embase and PsycInfo—using the following search terms: (“Obesity”[All Fields] OR “Adiposity”[All Fields]) AND (“Mild cognitive impairment”[All Fields] OR “Cognitive decline”[All Fields] OR “Dementia”[All Fields]) AND (“Artificial intelligence”[All Fields] OR “Machine learning”[All Fields]) (Table S1). We conducted independent scans of titles and abstracts from database searches. Articles were evaluated based on predetermined inclusion criteria to determine their suitability. All articles that met the inclusion criteria for the full text were selected based on their titles and abstracts.

This research was not restricted by the year of publication for the articles considered.

Inclusion criteria: (i) articles that enrolled human subjects; (ii) experiments that focused on the relation between cognitive impairment and obesity in adults; (iii) articles that used AI or ML techniques; (iv) articles in English language only. Exclusion criteria: (i) reviews and meta-analyses; (ii) conferences and editorials; (iii) duplicated studies; (iv) animal studies; (v) non-English studies.

### 2.2. PICO Model

We employed the PICO (Population, Intervention, Comparison, and Outcome) model to shape our research question [41]. Our target population comprised midlife adult patients. The intervention was to investigate the utility of AI and ML in assessing the relationship between obesity and cognitive decline. For the comparison, we considered the traditional investigation methods and their utility in clinical practice in terms of costs and time. The outcome was related to the potential of AI and ML in addressing the interconnected issues of obesity, its associated health consequences, and cognitive decline.

### 2.3. Study Selection

A total of 200 articles were identified through database searches. In total, 25 duplicated articles were deleted; 16 reviews were removed; 12 editorials or conferences were deleted; 74 studies were removed after title screening; 57 were removed after reviewing abstracts; and 8 articles were removed after text screening (Figure 1). In this systematic review, we considered a total of 8 articles about obesity and cognitive decline with the use of ML techniques. To ensure the integrity of the study selection process, two authors extracted data independently (I.V. and A.G.), resolving any disagreements through collaborative discussion and consultation with a third author (G.M.). At least three authors independently evaluated each article, and in case of any disagreement, the other three authors were consulted. The necessary data were extracted from the full-text article. If any critical information was missing from the original studies, their authors were contacted. This method was implemented to eliminate the possibility of bias and to strengthen the validity and reliability of the study findings.

### 2.4. Data Extraction and Analysis

Following the full-text selection, data were extracted from the included studies and reported in a table using Microsoft Excel (Version 2021). The extracted data included study title, first author name, year of publication, study aims and design, sample size, type of participants, type of intervention and control, baseline performance, type of outcome and time points for assessment, results, and key conclusions. Moreover, the agreement between the two reviewers (I.V. and A.G.) was assessed using the kappa statistic. The kappa score, with an accepted threshold for substantial agreement set at >0.61, was interpreted to reflect excellent concordance between the reviewers. This criterion ensures a robust evaluation of inter-rater reliability, emphasizing the achievement of a substantial level of agreement in the data extraction process.

### 2.5. Risk of Bias within Individual Studies

I.V., A.G., and G.M. independently assessed the risk of bias for each study, which was cross-checked by L.C. The risk of bias was assessed using the Risk of Bias in Non-Randomized Studies of Exposure (ROBINS-E) (2023) tool [42] that comprises seven domains: (i) bias due to confounding; (ii) bias arising from the measurement of the exposure; (iii) bias in the selection of participants for the study (or for the analysis); (iv) bias due to post-exposure interventions; (v) bias due to missing data; (vi) bias arising from the measurement of the outcome; and (vii) bias in the selection of the reported results.

## 3. Results

### 3.1. Synthesis of Evidence 

We conducted a systematic review to investigate the relationship between obesity, its consequences, and cognitive decline. In total, we analyzed eight studies (Table 1): four of these utilized ML techniques for risk assessment and prediction, while the other four employed ML to understand mechanisms and identify biomarkers.

### 3.2. Key Findings from Included Studies

These recent studies have revealed more about the intricate connections between dementia, cognitive decline, and the multiple factors that contribute to these conditions. The relationships between these elements are not straightforward but rather involve interrelated factors that interact in complex ways to influence cognitive health. These factors could include genetics, lifestyle choices, environmental factors, and medical conditions [43]. Despite using different methods, these studies collectively provide valuable insights into this critical health research area. Emphasizing the connection between these conditions, research has shown that even mild hyperglycemia can lead to faster cognitive decline in individuals under 88 years of age who also have central obesity [44]. Conversely, among those under 87 years of age without central obesity, adiponectin may be an independent risk factor for cognitive decline [44]. Additionally, the “Likely Dementia” status is more prevalent among older individuals, with a 2:1 female-to-male ratio, and is associated with nine factors that increase the risk of transitioning to dementia. These factors include low levels of education, hearing loss, hypertension, drinking, smoking, depression, social isolation, physical inactivity, diabetes, and obesity [45]. Several studies have highlighted the differential impact of sex on dementia risk [46,47]. For example, a study by Plassman (2007) [48] found that women tend to have a higher prevalence of dementia compared to men, partly due to longer life expectancy but also potentially influenced by biological and sociocultural factors. Other studies have also corroborated a greater incidence of AD among women, with longevity remaining a significant contributing factor to the higher prevalence of AD among females compared to males [49,50]. Research also suggests that there is a direct correlation between BMI trajectories and diabetes, hypertension, and dementia [51]. Additionally, ML has been employed to predict dementia based on modifiable risk factors, indicating the potential for preventive intervention. These ML models have identified BMI, cognitive activity, and physical activity as the most important features for predicting the risk of dementia [52]. Apart from identifying risk factors, some of these studies delve deeper into the underlying mechanisms. For instance, ML was utilized to quantify the impact of cardiovascular and metabolic risk factors (CVMs) on brain structure, providing valuable insights into how these factors influence cognitive decline. In a recent study [53], CVMs, such as diabetes and hypertension, had the strongest association with changes in brain structure, suggesting a cumulative effect. Interestingly, ML-based index scores did not show any correlation with established markers for brain aging or AD, implying that these factors have distinct effects from typical age-related changes and dementia [53]. Furthermore, health-disease phase diagrams (HDPDs) were introduced to visually represent an individual’s risk progression for various diseases, including dementia. This can aid in early detection and personalized interventions, helping to improve health beyond personal boundaries [54]. In fact, HDPDs can potentially prevent future disease onset in 7 out of 11 diseases [54]. Distinct patterns have been revealed in how metabolic health relates to brain integrity in men and women. Research shows that men may be more vulnerable to the direct consequences of metabolic health on brain damage and cognitive decline. On the other hand, women’s cognitive decline may be associated with more complex interactions that involve brain health, metabolism, and possibly genetics [55]. This highlights the need for personalized approaches to address the unique needs of each individual. Lastly, ML was also used to identify potential diagnostic genes for both AD and MetS, suggesting common underlying mechanisms. A cluster of genes related to cellular processes such as organization and transport were identified with the potential to diagnose both AD and MetS [56].

**Table 1:** Characteristics of the studies included (by year).

Study	Design	Sample Size	AI/ML Technique	Cognitive Decline and Assessment	Objective	Results
Bin-Hezam and Ward (2019) [52]	Secondary observational	1812 subjects (aged 55–90) from ADNI.	LR, NB, DT, and RF	This study analyzes existing diagnoses and dementia risk factors within the ADNI dataset (e.g., depression, cognitive inactivity).	Detect dementia based on modifiable risk factors leveraging ML techniques.	ML models achieved high accuracy (up to 92%) in predicting dementia risk. BMI, cognitive, and physical activity were identified as important factors.
Ganguli et al., 2020 [44]	Prospective cohort	478 individuals aged 65 and older from MYHAT	WHR-stratified ML analyses using CART	Neuropsychological tests for memory, attention, and other cognitive functions were administered at baseline and follow-up to assess global cognitive decline.	Investigate potential underlying mechanisms between diabetes, obesity, and cognitive decline in older adults.	Hyperglycemia in younger individuals with central obesity may lead to faster cognitive decline, while adiponectin could be a risk factor for cognitive decline in younger individuals without central obesity.
Foret et al. (2021) [55]	Observational	266 individuals (121 males and 145 females; mean age ± SD: 49 ± 6 years for both)	Gaussian process	Standardized memory, fluency, and executive function tests were administered (e.g., MMSE, CVLT-II, WAIS-IV) to assess late-life cognitive decline.	Investigate the relationships between brain health and cardiovascular risk factors in midlife adults, with a specific focus on sex differences.	Men might be more vulnerable to the direct effects of metabolism on brain health, while women might experience more complex interactions involving brain health, metabolism, and potentially genetics.
Govindarajan et al. (2022) [53]	Cross-sectional observational study	N = 24,902 from 8 independent studies, 54.5% female, average age = 62.4, age range 45–75 years.	Linear support vector classifiers	This study analyzes existing data (diagnoses and MRI scans) from cognitively normal iSTAGING participants to develop CVM-related brain structure measures (SPARE-CVMs) relevant to dementia, AD, and brain aging.	Develop and evaluate ML-based indices that can capture the individual-level effects of CVMs on brain structure in cognitively unimpaired individuals.	ML successfully revealed distinct brain changes associated with specific CVMs like diabetes and hypertension in cognitively normal individuals. These indexes captured distinct patterns and were not linked to typical aging or AD markers, highlighting their potential for understanding CVM-brain health relationships.
Li et al. (2022) [56]	Observational	Data of AD and MetS in the GEO database	RF and LASSO	The study focuses on gene expression data from existing studies of AD and MetS.	Explore the connection between AD and MetS by identifying genes that could potentially diagnose both conditions.	The study found 8 genes potentially useful for diagnosing both AD and MetS.
Gharbi-Meliani et al. (2023) [45]	Longitudinal observational	Data of 15,278 baseline participants (aged 50 years and more) from SHARE	MFA and HCPC	Autonomy was assessed via ADL/IADL scales; cognition was assessed via immediate recall and VF for “Likely Dementia” status.	Explore the potential of unsupervised ML in identifying transition to probable dementia in longitudinal population aging surveys.	“Likely Dementia” is more common in older people, with a higher prevalence in females than males. Nine risk factors including hypertension, physical inactivity, diabetes, and obesity, increase the likelihood of transitioning to dementia.
Mottalib et al. (2023) [51]	Observational cohort study	Data from 1,531,374 patients (aged 20–70) collected from 1 January 2013 to 31 December 2018	Unsupervised clustering	Focused on individuals having a diagnosis of one of the 18 major chronic diseases examined, including AD or dementia.	Classify the likelihood of 18 common chronic illnesses in individuals based solely on their obesity patterns, as indicated by the trajectory of their BMI.	The development of diabetes, hypertension, and dementia is directly related to the trajectory of BMI.
Nakamura et al. (2023) [54]	Observational cohort study	Data from 3238 individuals (1281 males, age 50.2 ± 16.2 years; 1957 females, age 51.5 ± 16.0 years) collected over 14 years (2005–2018).	Future-onset prediction models and p-mICE	11 NCDs were considered, including incident dementia that was defined by the MMSE ≤ 23.	Investigate the potential of HDPDs for early detection and prevention of NCDs.	HDPDs revealed individual variations in health markers, suggesting personalized prevention strategies. Improving health beyond personal boundaries in HDPDs potentially prevented future disease onset in 7/11 diseases.

Legend: ADNI = Alzheimer’s Disease Neuroimaging Initiative; LR = logistic regression; NB = naive Bayes; DT = decision tree; RF = random forest; ML = machine learning; BMI = body mass index; MYHAT = Monongahela–Youghiogheny Healthy Aging Team; WHR = waist–hip ratio; CART = classification and regression tree; CVLT-II = California Verbal Learning Test-II; MMSE = Mini-Mental State Examination; WAIS-IV = Wechsler Adult Intelligence Scale-IV; MRI = magnetic resonance imaging; CVMs = cardiovascular and metabolic risk factors; AD = Alzheimer’s disease; MetS = metabolic syndrome; GEO = Gene Expression Omnibus; LASSO = least absolute shrinkage and selection operator; SHARE = Survey of Health, Ageing, and Retirement in Europe; MFA = multiple factor analysis; HCPC = hierarchical clustering on principal components; ADL = activities of daily living; IADL = instrumental activities of daily living; VF = verbal fluency; p-mICE = partially modified individual conditional expectation; NCDs = noncommunicable diseases; HDPDs = personalized health-disease phase diagrams.

### 3.3. Risk of Bias

**Figure 2:**
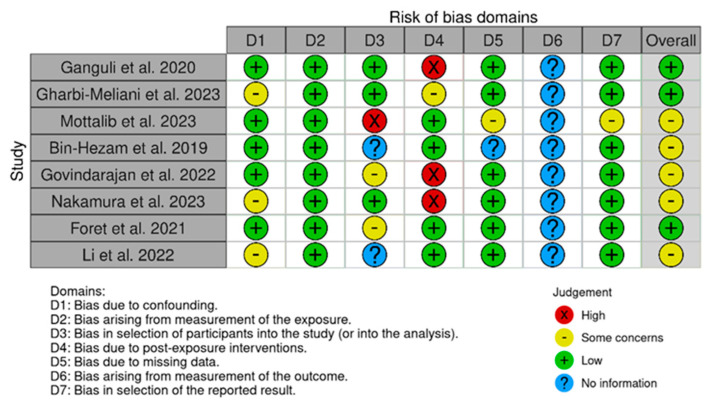
Risk of bias of the included studies (ROBINS-E) [44,45,51,52,53,54,55,56].

The ROBINS-E tool [42] was used to assess the risk of bias in the articles included in this review. Figure 2 shows the summary of the risk of bias assessment, while the graphs depict the distribution of bias concerns across the included studies. Out of the total studies assessed, three studies [44,53,54] showed a high risk of bias due to post-exposure interventions, and one [51] reported a high risk of bias in the selection of participants for the study (or for the analysis). Additionally, three studies [44,53,56] displayed some concerns about bias due to confounding. Further, the studies conducted by Govindarajan et al. (2022) [53] and Foret et al. (2021) [55] exhibited some concerns about bias in the selection of participants for the study (or for the analysis). Moreover, some concerns about bias arising from the measurement of the exposure were found in the study of Gharbi-Meliani et al. (2023) [45]. Finally, another study [51] showed some concerns about the selection of participants and the selection of the reported results.

## 4. Discussion

This systematic review explored the potential of AI and ML in tackling the interconnected issues of obesity, its associated health consequences, and cognitive decline. Examining the genetic underpinnings of obesity, genome-wide association studies (GWAS) have uncovered over two hundred loci consistently linked with BMI, obesity prevalence, and fat distribution metrics [57]. There are several potential biological mechanisms through which obesity may increase the risk of cognitive impairment. The most apparent mechanism involves an increased risk of stroke, diabetes, and cardiovascular disease, as these conditions are known to heighten cognitive impairment risk and are often linked with obesity [58,59]. Further, insulin resistance associated with obesity is linked to a systemic chronic low-grade inflammatory response. Regrettably, the brain is not exempt from these inflammatory effects, which can ultimately accelerate the progression of cognitive decline and increase the risk of developing AD [60]. Obesity may be associated with higher levels of proinflammatory cytokines (like interleukin-1β) in the brain [61], which may explain why obesity remains linked to executive function even after accounting for blood pressure (BP) and fasting plasma glucose (FPG) [62]. Neuroinflammation related to obesity and activation of microglia induces synaptic remodeling and neuronal apoptosis and decreases neurogenesis, which has been associated with cognitive decline [63,64,65]. Additionally, research has shown a connection between obesity in early old age and a decrease in cortical thickness [66]. These findings collectively suggest that obesity and its associated metabolic problems may contribute to increased microvascular brain damage and neuroinflammatory events, ultimately leading to cognitive decline [62].

There might be inherent aspects of central adiposity that elevate cognitive impairment risk. Visceral adipose tissue is a metabolically active endocrine organ that secretes numerous inflammatory cytokines and hormones [67,68]. Research suggests that leptin can cross the blood–brain barrier and may contribute to neurodegeneration [69,70]. Leptin is also implicated in the deposition of amyloid beta 42, the primary component of AD-associated plaques in the brain [71]. Recent research discovered that obese middle-aged adults exhibit reduced brain volume compared to those of normal weight [72], while another study found that high central obesity in elderly adults was linked to decreased hippocampal brain volume and increased brain atrophy [73].

We analyzed eight studies, revealing a complex relationship between these conditions, with factors like central obesity, hyperglycemia, and hypertension playing significant roles [44,45,51,53]. Statistical methods used may provide valuable insights into these associations but cannot establish cause and effect due to data restrictions and limited generalizability of findings [44]. Another approach shows promise as it identifies potential cases for further investigation without requiring pre-existing diagnoses [45]. However, it relies on the “Likely Dementia” designation, which needs clinical confirmation and does not differentiate between dementia subtypes [45]. AI and ML have still emerged as valuable tools for both risk assessment and prediction. These models excel in assessing risk and predicting cognitive decline in patients with obesity due to their ability to glean valuable insights from data. By analyzing vast datasets, they can identify complex patterns and subtle connections between obesity, health factors, and cognitive decline that traditional methods might overlook. This has allowed researchers to identify key risk factors like BMI, physical activity levels, and cognitive activity in predicting dementia risk [51,52]. However, the k-means clustering technique might not be the most advanced tool for analyzing complex time-series data, and a focus on BMI alone may exclude other factors that could influence chronic disease risks [51]. ML techniques can even delve deeper, exploring the biological mechanisms at play, such as the impact of cardiovascular and metabolic factors on brain structure [53,55,56]. However, these models can be complex and hard to interpret due to their “black box” nature, requiring specialized knowledge. It is important to note that both traditional and ML approaches heavily depend on the quality of the data they receive. Poor data quality may result in crucial aspects of brain health being overlooked, leading to an incomplete understanding of the intricate web of factors impacting brain health. Early risk identification through ML models allows for timely interventions, potentially mitigating the severity of both obesity and cognitive decline [54]. The study by Nakamura et al. (2023) [54] provides HDPDs for the early detection and prevention of various NCDs, including dementia. These findings suggest personalized prevention strategies, which could potentially prevent the onset of future diseases in 7 out of 11 cases [54]. Nonetheless, limiting the analysis of data to a single location with a healthy population can lead to a reduced number of participants who develop specific health conditions. For instance, the time-series analysis of some NCDs did not reveal any significant difference, which might be partially attributed to the small number of records of patients with dementia [54]. Furthermore, while the retrospective approach employed in the study provides valuable insights, it does not conclusively establish the effectiveness of interventions based on model predictions. To solidify the clinical usefulness of the model, prospective studies that track the outcomes of the interventions designed around these predictions would be necessary. Gamification and interactive learning elements could further enhance user engagement, making the process of behavior change more enjoyable and sustainable. Seamless integration with wearable technology would allow for real-time data collection and feedback, enabling the platform to continuously adapt recommendations based on individual progress. Further studies are needed to explore how AI and ML can be used to create personalized interventions tailored to individual needs and risk factors, encompassing diet, exercise, and cognitive stimulation activities. This review emphasizes the importance of personalized approaches due to observed sex differences in how metabolic health affects brain integrity. While men might be more susceptible to the direct consequences of metabolic health on the brain, women might experience a more complex interplay involving brain health, metabolism, and possibly genetics [56]. Importantly, these technologies can pave the way for personalized medicine by tailoring risk assessments, prevention strategies, and even treatment plans to each patient’s unique needs, considering sex differences in how metabolic health affects the brain. Female patients diagnosed with dementia have a higher correlation with CVD risk factors compared to male patients [74]. Meanwhile, male patients tend to have a higher correlation with behavioral risks and other factors [75]. It is now widely accepted that vascular risk factors that increase the chances of heart disease, such as hypertension, diabetes, obesity, and hyperlipidemia, also compromise cerebrovascular health [76]. It is crucial to detect and control these modifiable risk factors that exist throughout a person’s lifetime, as managing them early on is currently the only known prevention method [77]. Therefore, there is a pressing need for more efficient strategies to combat multimorbidity and speed up early diagnosis and management [78].

### 4.1. Future Directions

This review highlights the need for further research to explore the potential of AI and ML in developing more accurate and personalized predictive models. This will help in designing and evaluating effective interventions based on AI and ML insights. While the reviewed studies shed light on the potential of AI and ML in understanding the link between obesity and cognitive decline, some notable gaps remain in the literature. The absence of automatic telemedicine models is striking. These models could offer comprehensive care by monitoring health parameters, providing psychological support, and even assessing the risk of cognitive decline in obese patients. Their development would represent a significant advancement in preventative care. For instance, AI/ML tools could be developed to assess food intake more effectively using image recognition or personalized dietary surveys. These data, in combination with other health markers, could be used by AI/ML models to identify individuals at risk of obesity and cognitive decline at an early stage, allowing for preventative interventions [79,80]. Secondly, the studies primarily focus on risk prediction, with limited exploration of intervention design. According to the research findings [54], it would be interesting to develop sex-specific AI and ML models for more accurate predictions and personalized interventions. AI can also assist healthcare professionals in their decision-making processes and analyze patient data to help stratify risk and recommend personalized treatment plans for cognitive decline in obese patients [81]. ML can analyze vast datasets to identify the most effective treatment strategies for individual cases, considering various factors like patient characteristics and potential side effects. Additionally, AI can optimize resource allocation within the healthcare system by identifying individuals who would benefit most from different levels of intervention. Future research should also explore cost-effective ways to implement AI/ML solutions in healthcare settings. Although initial costs may be a concern, AI/ML models have the potential to identify high-risk individuals and optimize resource allocation, which could lead to long-term cost savings. These are just a few examples, highlighting the vast and ever-evolving potential of AI and ML in addressing the challenges of obesity, cognitive decline, and other related health concerns.

### 4.2. Limitations

This systematic review highlights the potential of AI and ML in addressing the concerning link between obesity and cognitive decline. Eight studies were chosen through a comprehensive search strategy across various databases. To ensure transparency and eliminate bias, clear inclusion and exclusion criteria were established. The review acknowledges that limitations exist due to the included studies, representing a relatively new and evolving field. The methodologies employed across the studies varied considerably. This diversity is likely due to the ongoing development of AI and ML applications in this area. These factors, combined with the limited number of studies, indicate that further research is necessary to improve the applicability of these findings.

## 5. Conclusions

Evaluating the effectiveness of AI and ML interventions is crucial to assess their real-world impact. Early detection, prevention, and personalized interventions through these technologies could lead to substantial cost savings by minimizing the need for expensive treatments and long-term care. Additionally, AI-powered tools can streamline healthcare delivery, making preventive care more accessible and cost-effective. However, realizing these benefits requires careful consideration of ethical concerns, ensuring data privacy, addressing potential biases, and guaranteeing equitable access for all. While the initial development and implementation of these technologies can be expensive, their long-term potential for cost reduction and time saved in the fight against obesity and cognitive decline is significant. As research continues to advance, we can expect to see even more innovative and impactful applications of these technologies emerge in the years to come.

## Data Availability

Data are contained within the article and Supplementary Materials.

## Supplementary Materials

The following supporting information can be downloaded at: https://www.mdpi.com/article/10.3390/jcm13082307/s1, Table S1: Syntax authors’ searches in various databases.
