# Supplementary material for: Towards a Deeper Understanding: Utilizing Machine Learning to Investigate the Association between Obesity and Cognitive Decline—A Systematic Review
Source: J Clin Med. 2024 Apr 16;13(8):2307. doi: 10.3390/jcm13082307 (PMC11051247; doi:10.3390/jcm13082307)
Supplement: Supplementary file 1 [file jcm-13-02307-s001.zip › jcm-2946173-supplementary.pdf]

Table S1: Syntax authors' searches in various databases.

Database	Search String	N of Results
Scopus	ALL ("OBESITY" AND "COGNITIVE DECLINE" AND "DEMENTIA" AND "MILD COGNITIVE IMPAIRMENT" AND "ALZHEIMER’S DISEASE" AND "ARTIFICIAL INTELLIGENCE" AND "MACHINE LEARNING")	52
Pubmed	("Obesity"[All Fields] OR "Adiposity"[All Fields]) AND ("Mild cognitive impairment"[All Fields] OR "Cognitive decline"[All Fields] OR "Dementia"[All Fields]) AND ("Artificial intelligence"[All Fields] OR "Machine learning"[All Fields])”	24
Embase	('obesity'/exp OR adiposity) AND ((mild AND cognitive AND 'impairment'/exp OR cognitive) AND 'decline'/exp OR 'dementia'/exp) AND (artificial AND 'intelligence'/exp OR machine) AND 'learning'/exp	4
Web of Science	TS=(obesity OR adipose tissue) AND TS=(mild cognitive impairment OR cognitive decline OR dementia) AND TS=(artificial intelligence OR machine learning)	25
PsycInfo	obesity AND Any Field: cognitive decline OR Any Field: dementia AND Abstract: machine learning OR Abstract:	94

	artificial intelligence AND Population Group: Human AND Peer-Reviewed Journals only	
Cochrane	OBESITY in Keyword AND COGNITIVE DECLINE in Keyword OR DEMENTIA in Keyword AND ARTIFICIAL INTELLIGENCE in Keyword AND MACHINE LEARNING in Keyword	1
